# Genetic identification of the selenate reductase in *Enterobacter cloacae* SLD1a-1

**DOI:** 10.1128/aem.01796-25

**Published:** 2025-11-26

**Authors:** Jonathan Phan, Dylan Klein, Vikas Nanda, Gerben Zylstra, Nathan Yee

**Affiliations:** 1 Department of Environmental Sciences, Rutgers University 5970, New Brunswick, New Jersey, USA; 2 Department of Biochemistry and Microbiology, Rutgers University 5970, New Brunswick, New Jersey, USA; 3 Department of Biochemistry and Molecular Biology, Robert Wood Johnson Medical School, and Center for Advanced Biotechnology and Medicine, Rutgers University 242612, Piscataway, New Jersey, USA; 4 Department of Earth and Planetary Sciences, Rutgers University 5970, Piscataway, New Jersey, USA; Georgia Institute of Technology, Atlanta, Georgia, USA

**Keywords:** DMSO reductase, molybdopterin guanine dinucleotide, *ynfE*, *dmsD*, anaerobic respiration

## Abstract

Bacterial selenate reduction is a key microbial process that affects the speciation and mobility of selenium in the environment. In this study, we identified the selenate reductase gene in the facultative anaerobe *Enterobacter cloacae* SLD1a-1. Genome sequencing revealed a membrane-bound, twin-arginine translocation (TAT) exported molybdoenzyme operon designated as *srnABCD*, under the regulation of the fumarate and nitrate reductase regulator (FNR) transcription factor. The *srnA* gene encodes a molybdenum-containing subunit; *srnB* and *srnC* encode iron-sulfur and membrane anchor subunits, respectively; and *srnD* encodes a TAT chaperone. Targeted mutagenesis of the *srnA* gene resulted in a mutant defective in selenate reduction. Complementation with the wild-type *srnA* sequence restored the abolished phenotype. Heterologous expression of *srnA* in an *Escherichia coli* Δ*ynfEF* mutant conferred selenate reduction activity, demonstrating cross-species functionality. Protein structure modeling of the selenate reductase using Boltz-1 showed a funnel-shaped active site involved in selenate binding and reduction. These findings provide new molecular insights into the genetics and mechanism of bacterial selenate reduction.

**IMPORTANCE:**

Selenium pollution poses risks to ecosystems and human health, largely due to the mobility and toxicity of selenate, a common form found in soil and water. Diverse bacterial species are able to convert soluble selenate into insoluble elemental selenium, but the genes and enzymes that underpin this process are not fully understood. In this study, we identified a gene in *Enterobacter cloacae* SLD1a-1 that enables the bacterium to catalyze selenate reduction. We showed that this gene produces a functional enzyme even when it is transferred into a different species, *Escherichia coli*. Protein structure modeling revealed features of the enzyme that help it recognize and reduce selenate. This information advances our understanding of how selenium is enzymatically cycled in the environment.

## INTRODUCTION

Diverse microorganisms have evolved the ability to catalyze dissimilatory selenate [Se(VI), SeO_4_^2−^] reduction (1, 2). Se(VI) can be used as an alternate terminal electron acceptor for anaerobic respiration yielding more energy than sulfate or Fe(III) reduction under standard conditions (2, 3). Microorganisms capable of dissimilatory selenate reduction span across multiple phyla and have been isolated from a wide range of environments, including estuary sediments, soda lakes, contaminated soils, and hydrothermal waters (4–8). Because microbial reduction of soluble SeO_4_^2−^ oxyanions forms insoluble elemental selenium [Se(0)] in anoxic sediments (9, 10), Se(VI) respiration can control the fate and removal of dissolved selenium in aquatic ecosystems (11). Despite the ubiquity and ecological relevance of Se-reducing bacteria, the molecular basis of Se(VI) respiration remains poorly understood, with only a few reductases functionally characterized. The most well-studied is the periplasmic selenate reductase SER in *Thauera selenatis* (12–14). A dissimilatory membrane-bound selenate reductase SRD has also been characterized in the Gram-positive bacterium *Mesobacillus* (formerly *Bacillus*) *selenatarsenatis* SF-1 (15). The genes that encode these enzymes are distantly related, suggesting that bacterial selenate reductases have multiple evolutionary origins.

*Enterobacter cloacae* SLD1a-1 is a Se(VI)-reducing facultative bacterium isolated from agricultural drainage water in the San Joaquin Valley, California (16). In nitrate-depleted anaerobic environments, *E. cloacae* relies on selenate respiration to sustain cellular maintenance, gaining a selective advantage through the generation of proton motive force via its membrane-bound selenate reductase (17). Selenate reduction in *E. cloacae* is mediated by the anaerobic electron transport chain, specifically requiring menaquinone as an electron donor to the terminal selenate reductase (18). The selenate reductase is regulated by the fumarate and nitrate reductase regulator (FNR), an oxygen-sensing transcription factor that activates the expression of the selenate reductase gene under anaerobic conditions (19). Se(VI) reduction is catalyzed by a molybdenum-dependent membrane-bound enzyme that is distinct from the nitrate reductase, localized to the cytoplasmic membrane with its active site facing the periplasm (20). The specific reaction catalyzed by the selenate reductase is:


$$SeO_{4}^{2-}+2H^{+}+2e^{-}↔SeO_{3}^{2-}+H_{2}O\;\;E_{O}^{\prime }=+0.48\;\text{V}$$

Export of the selenate reductase to its active location depends on the twin-arginine translocation (TAT) system (21). Biochemical studies have shown that the enzyme is a heterotrimeric complex with subunits of ~100, ~55, and ~36 kDa (22). Currently, the genes encoding this protein complex are unknown.

In this study, we sequenced the genome of *E. cloacae* SLD1a-1 and conducted gene knockout experiments to identify the selenate reductase gene. The objectives were (i) to search the genome for a membrane-bound TAT targeted molybdoenzyme under the regulation of FNR, (ii) to determine if the putative selenate reductase gene is required for selenate reduction, and (iii) to validate the function by expressing the selenate reductase gene in an *Escherichia coli* mutant strain lacking its native selenate reductase. The results demonstrated the genetic basis of Se(VI) reduction in *E. cloacae* and yielded new insights into the molecular mechanism of the selenate reductase.

## RESULTS AND DISCUSSION

### Identification of the selenate reductase gene in *E. cloacae*

The draft genome sequence of *E. cloacae* SLD1a-1 contained 4,874,235 base pairs with a G+C content of 54.9% (Table S1). A total of 4,601 genes were predicted. Hmmsearch of the genome with the Pfam HMM database revealed 4,199 proteins with Pfam domains, including 13 unique proteins with a Pfam domain for molybdopterin dinucleotide binding. Signal peptide prediction identified 571 proteins with signal motifs, of which 19 contained a TAT signal sequence.

**Fig 1:**
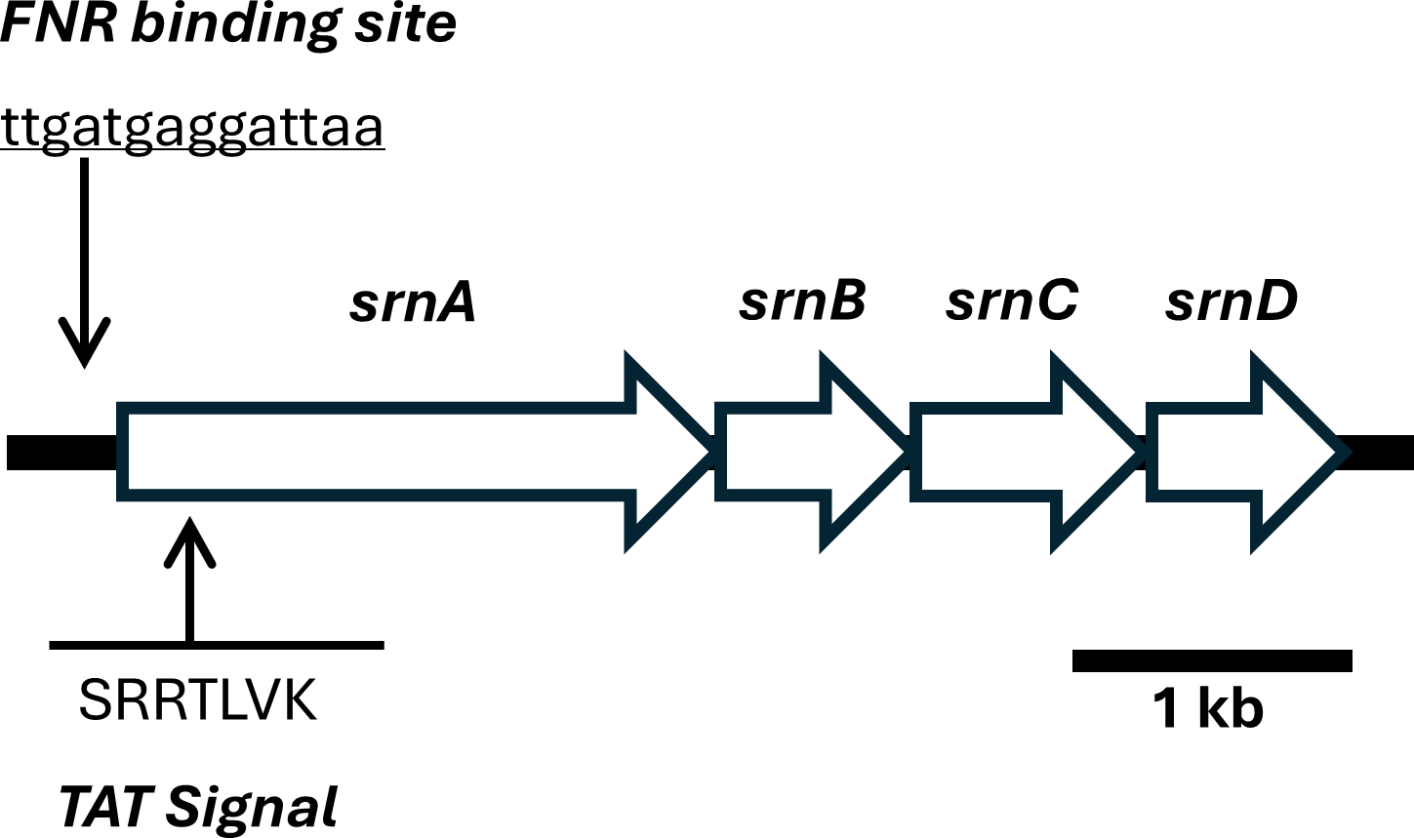
Gene map of the *srnABCD* operon encoding the selenate reductase in *E. cloacae* SLD1a-1. The top arrow indicates a FNR binding site located upstream of the operon. The bottom arrow marks the TAT signal sequence found within the N-terminus of the *srnA* gene product.

A TAT-targeted molybdopterin dinucleotide binding protein under the regulation of FNR was identified in the genome sequence (Fig. 1). The putative selenate reductase gene was found in an operon consisting of four open reading frames (ORFs), which we designated as *srnABCD*. The *srnA* gene harbors a FNR binding site (ttgatgaggattaa) indicating the gene is expressed during the transition from aerobic to anaerobic respiration. The SrnA protein is predicted to bind a molybdenum cofactor, with the peptide containing a signal sequence (SRRTLVK) at the N-terminus for protein export via the TAT pathway. The adjacent gene *srnB* encodes for an iron-sulfur protein with cysteine residues that is expected to bind four iron-sulfur clusters. The gene product of *srnC* is a transmembrane protein that serves as a membrane anchoring subunit. The operon also contains *srnD*, which specifies a TorD-like TAT chaperone involved in protein maturation. Together, the sequence analysis indicated that the operon encodes for a TAT-dependent membrane-bound heterotrimeric protein complex, resembling the proposed selenate reductase in the facultative anaerobe *Citrobacter freundii* (23).

**Fig 2:**
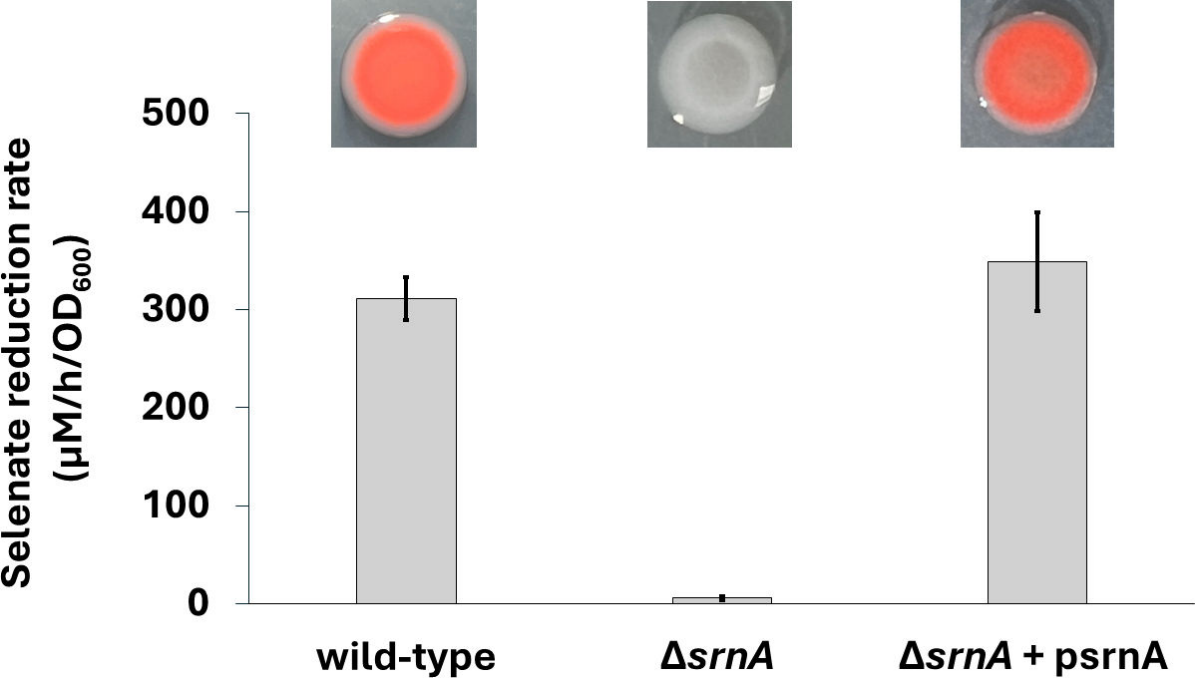
Se(VI) reduction in *E. cloacae* and Δ*srnA* mutant strains. The bar graph shows the selenate reduction rates measured for the wild-type strain (left), Δ*srnA* mutant (middle), and Δ*srnA* mutant strain complemented with psrnA (right). Error bars represent the standard deviation of triplicate experiments. Shown above are colonies of each strain grown on selenate-containing agar.

Gene knockout experiments showed that *srnA* is the selenate reductase gene in *E. cloacae* (Fig. 2). The *E. cloacae* wild-type strain reduced Se(VI) to Se(0) and precipitated red elemental selenium on selenate-containing LB agar plates. In liquid M9 medium, the wild-type strain reduced selenate at a rate of 311 ± 22 µM/hour/OD_600_. Mutation of *srnA* abolished selenate reduction in *E. cloacae*. A mutant strain containing an in-frame replacement of the *srnA* gene with *kanR* lost the ability to precipitate red elemental selenium and was unable to reduce selenate in liquid medium. Complementation of the Δ*srnA* mutant with the plasmid psrnA containing the wild-type *srnA* gene fully restored the deletion mutant’s ability to reduce Se(VI) with a selenate reduction rate of 349 ± 51 µM/hour/OD_600_. The mutant retained selenite [Se(IV), SeO_3_^2−^] reduction activity (Fig. S1), demonstrating that Se(IV) reduction is mediated by a separate biochemical reaction.

Our data indicate that SrnA is the catalytic subunit of the membrane-bound heterotrimeric selenate reductase complex that was biochemically characterized by Ridley et al. (22). SrnA is the molybdenum-containing subunit that binds with iron-sulfur protein SrnB and membrane-anchoring subunit SrnC to form the functional selenate reductase in *E. cloacae*. The amino acid sequences for SrnB and SrnC lack identifiable signal sequences for protein translocation, thus suggesting they are not independently exported to the periplasm. Instead, these peptides likely bind with SrnA inside the cytoplasm to form a heterotrimer and are transported together through the TAT machinery as an assembled protein complex. One possible mechanism is that the folded domain of SrnAB passes through the TAT system, while the hydrophobic anchor SrnC is retained in the inner membrane during translocation. This would form a membrane-bound selenate reductase complex with its active site facing the periplasm (20).

### Heterologous expression of *srnA* in *E. coli*

**Fig 3:**
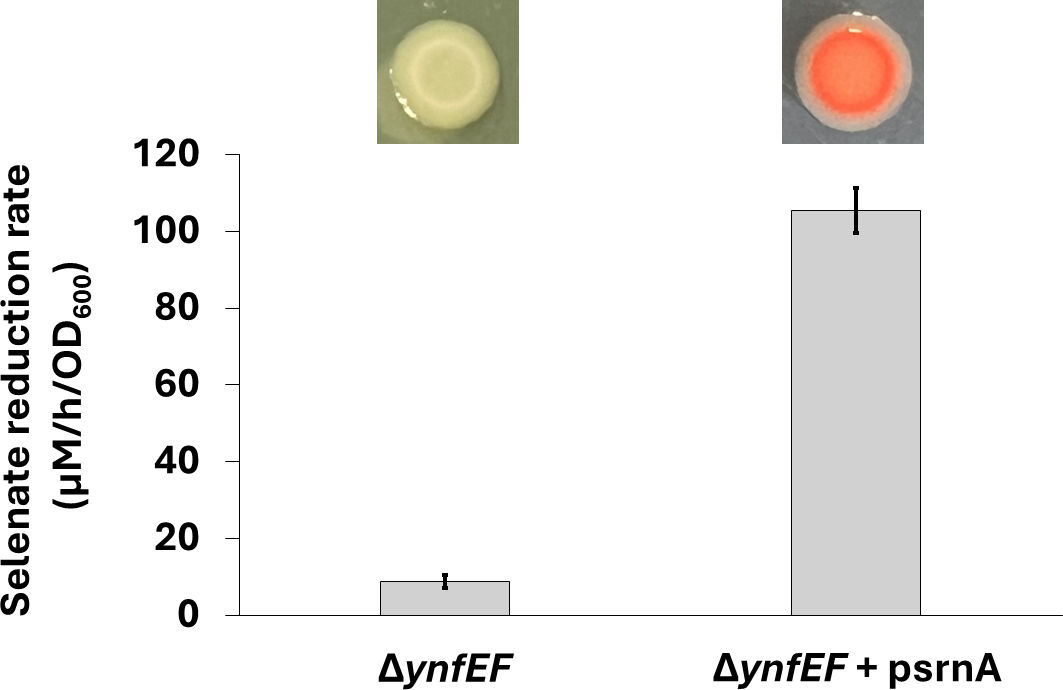
Heterologous expression of *srnA* in *E. coli*. The bar graph shows selenate reduction rates for the *E. coli* Δ*ynfEF* mutant (left) and the Δ*ynfEF* mutant complemented by psrnA (right). Error bars represent the standard deviation of triplicate experiments. Shown above are colonies grown on selenate-containing agar.

To test if the *srnA* gene is functional in *E. coli*, we conducted a heterologous expression experiment with an *E. coli* Δ*ynfEF* mutant. The wild-type *E. coli* strain contains the operon *ynfEFGH-dmsD* where YnfE and YnfF are paralogous selenate reductase isoenzymes (24). Deletion of the native *E. coli* selenate reductase gene pair *ynfEF* resulted in a mutant strain that was unable to reduce Se(VI) to Se(0) (Fig. 3). Selenite reduction activity was unaffected (Fig. S1). Complementation of the *E. coli* Δ*ynfEF* mutant with the *srnA* gene from *E. cloacae* restored selenate reduction in the *E. coli*. The *E. coli* Δ*ynfEF* mutant harboring the plasmid psrnA expressed a functional reductase and reduced selenate at a rate of 110 ± 7 µM/hour/OD_600._ When cultured on selenate-containing agar, the complemented *E. coli* Δ*ynfEF* mutant precipitated red elemental selenium.

The results indicate that *srnA* encodes a functional selenate reductase gene in *E. coli*. Cross-species functionality of *srnA* is likely due to the fact that the *ynfEFGH-dmsD* operon in *E. coli* encodes a TAT-dependent selenate reductase system that parallels the organization and function of *srnABCD*. Similar to the assembly mechanism proposed for SrnABC protein complex, we posit that SrnA binds with the *E. coli* YnfG iron-sulfur subunit and the YnfH membrane-anchor subunit to form a functional heterotrimeric selenate reductase. Previously, we demonstrated that menaquinone serves as the electron donor that drives selenate reduction in *E. coli* (18). Once the protein complex is embedded in its active location, the YnfH membrane protein is the subunit that interacts with electron carriers in the inner membrane respiratory chain. Interestingly, the *ynfEFGH-dmsD* operon in *E. coli* contains a TAT chaperone gene *dmsD* that has been experimentally shown to be required for selenate reduction (24). Although DmsD shares only 69.1% sequence identity with SrnD, the TAT chaperone in *E. coli* appears to be able to interact with SrnA for protein maturation and functional conservation across species.

### Protein structure

**Fig 4:**
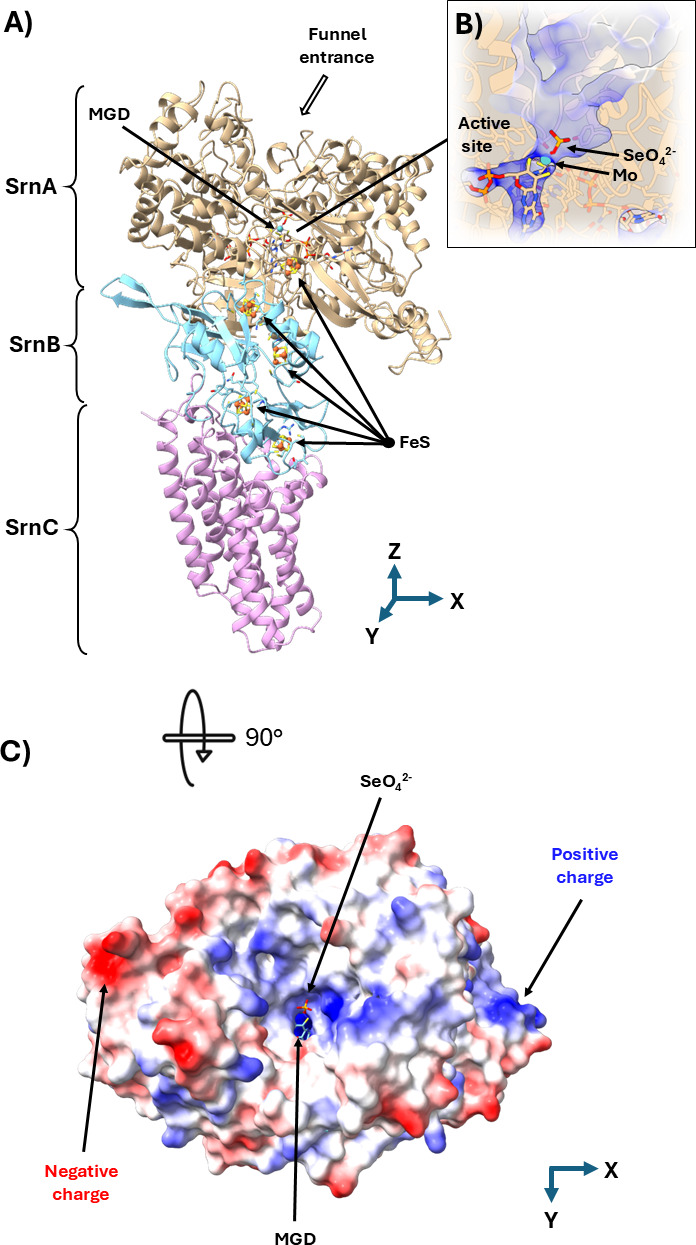
Structural model of the SRN protein complex and the surface architecture of the catalytic subunit. (**A**) The selenate reductase heterotrimer with the catalytic subunit SrnA (pale brown), the iron-sulfur SrnB subunit (light blue), and the membrane anchor SrnC (magenta). The molybdenum atom coordinated by two molybdopterin guanine dinucleotides is labeled as MGD, and the iron-sulfur clusters are labeled as FeS. At the top of the protein, the funnel entrance is indicated by an arrow. (**B**) A schematic cross-section of the funnel illustrating a channel to the active site with the selenate anion (yellow selenium, red oxygen) interacting with the molybdenum cofactor. Regions of positive charge guiding the SeO₄²^−^ anion into the catalytic pocket are shown in blue. (**C**) Surface representation of SrnA rotated 90° along the horizontal axis. A bird’s-eye view looking down the SrnA funnel entrance shows the active site where the selenate anion is positioned for reduction by the molybdenum cofactor. Positive charge is displayed in blue and negative charge in red.

To gain new insights into the biochemistry of the selenate reductase, the three-dimensional protein structure of SRN was modeled using Boltz-1 and AlphaFold3 (Fig. 4 and Fig. S2). The two models converge in atomic detail on the same protein domain arrangement and cofactor configurations. Boltz-1 yielded a high confidence protein structure prediction and assembled the three subunits into a heterotrimeric complex (Fig. 4A). Inter-subunit contacts were observed, with SrnA interacting with SrnB, which was in turn bound to SrnC. In the SrnA subunit, Boltz-1 modeled a bis(MGD)-Mo(IV) complex, along with one [4Fe-4S] cluster at the N-terminus. SrnB was predicted to bind four [4Fe-4S] clusters, each coordinated by four cysteine residues. Iron-sulfur clusters are critical for selenate reductase function (25), and the [4Fe-4S] clusters in the *E. cloacae* protein complex are spatially arranged to form a molecular wire that extends from the molybdenum-containing active site in SrnA through the entire SrnB subunit. The locations of the iron-sulfur clusters are likely essential to the electron transport pathway, ensuring controlled and directional electron flow. The molecular wire terminates in SrnC, which serves as a membrane anchor unit that receives electrons from the menaquinone pool in the cytoplasmic membrane. There is no indication that SrnC contains heme iron, and neither the Boltz-1 nor AlphaFold3 predicted the involvement of cytochromes in the selenate reductase protein complex.

The protein folds of SrnA resemble the three-dimensional structure of the DMSO reductase in *Cereibacter* (formerly *Rhodobacter) sphaeroides* (26). The active site features a funnel-like structure, with the molybdenum cofactor positioned at the bottom of the cavity (Fig. 4B). Excess positive charge was found at the entrance of the funnel (Fig. 4C), which is analogous to the active site of the perchlorate reductase (27). As a negatively charged selenate oxyanion enters the positively charged channel, the anion descends to the base and binds to the molybdenum cofactor at the bottom of the funnel. Similar to the reaction mechanism for the DMSO reductase (26), we expect the reduction of Se(VI) to selenite [Se(IV)] to be concurrent with the oxidation of Mo(IV) to Mo(VI) in the cofactor. The reduction of selenite to Se(0) occurs at a separate location in *E. cloacae* (21) and is mediated by a different biochemical system.

### Comparison with other selenate reductases

The *srnABCD* gene cluster in *E. cloacae* shares similarities with the *serABDC* operon in *T. selenatis* (12). Both operons encode for selenate reductases that are exported to their active locations via the TAT pathway using TorD-like chaperones (e.g., SrnD and SerD). The catalytic subunits SrnA and SerA are molybdenum-containing enzymes connected to iron-sulfur proteins SrnB and SerB, respectively. An important difference is that the SRN complex in *E. cloacae* is a membrane-bound selenate reductase with SrnC functioning as a membrane-anchoring protein, whereas SER in *T. selenatis* is a periplasmic protein complex with SerC interacting with the electron transport chain.

**Fig 5:**
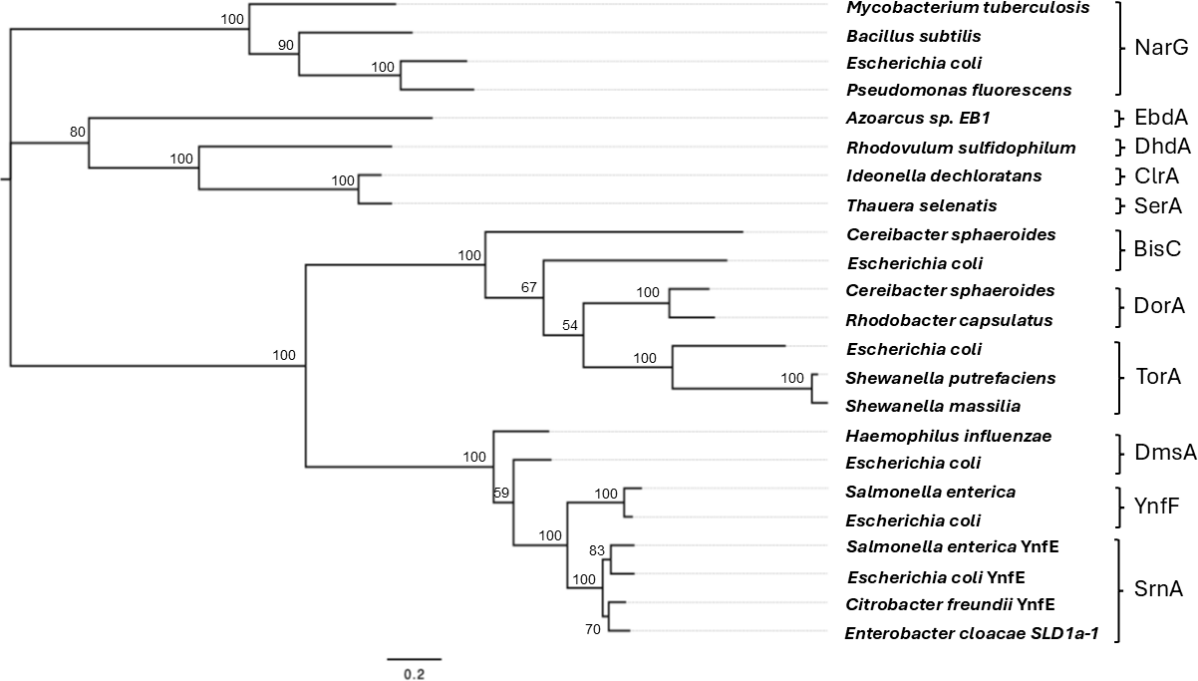
Maximum likelihood phylogenetic tree for representative sequences from the DMSO reductase family of molybdoenzymes related to the selenate reductases SrnA of *E. cloacae* SLD1a-1 and SerA of *T. selenatis*. Bootstrap values based on 500 replications are shown at branch nodes. Bar, 0.2% substitutions per position. NarG, nitrate reductase; EbdA, ethylbenzene dehydrogenase; DdhA, dimethylsulfide dehydrogenase; ClrA, chlorate reductase; SerA, selenate reductase; BisC, biotin sulfoxide reductase; DorA, DMSO/trimethylamine N-oxide reductase; TorA, trimethylamine N-oxide reductase; DmsA, DMSO reductase; YnfF, selenate reductase; YnfE, selenate reductase; SrnA, selenate reductase.

A comparison of the protein sequences indicates SrnA and SerA are distantly related, with only 24.3% sequence identity (Fig. 5). SrnA shares common ancestry with the dimethyl sulfoxide reductase DmsA (67.6% identity), while SerA is most closely related to the chlorate reductase ClrA in *Ideonella dechloratans* (83.6% identity). This is consistent with the hypothesis that bacterial selenate reductases have multiple evolutionary origins. We posit that this could be an example of convergent evolution where different enzymes have independently developed the ability to catalyze selenate reduction over billions of years of bacterial evolution.

SrnA is closely related to the selenate reductase YnfE of *C. freundii*, *E. coli*, and *Salmonella enterica* with sequence identities of 88.0%, 86.2% and 84.3%, respectively (Fig. 5). SrnA and these YnfE selenate reductases form a monophyletic group, sharing genetic similarity and common biological function. In the *E. coli* genome, “y” gene names are assigned to functionally uncharacterized ORFs (28). According to the systematic nomenclature guidelines described by Rudd (28), once a new function is established for an *E. coli* gene, the provisional “y” name should be abandoned, and a new gene name should be chosen (28). Based on the findings of this study and those of Guymer et al. (24), we recommend renaming the gene *ynfE* to *srnA*.

## MATERIALS AND METHODS

### Bacterial cultivation and growth conditions

The strains and plasmids used in the present study are listed in Table 1. All strains were grown at 37°C and maintained on Luria-Bertani (LB) agar unless otherwise mentioned. Antibiotics were added as supplements to the medium at the following final concentrations: ampicillin, 100 µg/mL; rifampicin, 100 µg/mL; tetracycline, 10 µg/mL; gentamicin, 25 µg/mL; and kanamycin, 50 µg/mL.

**TABLE 1:** Strains, plasmids, and primers

Strains, plasmids, and primers	Description	Reference
*E. cloacae* strains		
SLD1a-1	Wild-type strain, AmpR, RifR tagged	(16)
Δ*srnA*	Δ*srnA*, AmpR, RifR, KmR	This study
Δ*srnA* psrnA	Δ*srnA* harboring psrnA, AmpR, RifR, KmR, TetR	This study
*E. coli* strains		
BW25113	Wild-type; rrnB3 Δ*lacZ*4787 *hsdR514* Δ(*araBAD)567* Δ(*rhaBAD)568 rph-1*	(29)
Δ*ynfEF*	BW25113 Δ*ynfEF*	This study
Δ*ynfEF* psrnA	BW25113 Δ*ynfEF* harboring psrnA, TetR	This study
S17-1	Donor strain for conjugation	(30)
Plasmids		
pRK415	Broad host range expression vector; TetR	(31)
psrnA	pRK415 with *srnA* gene, TetR	This study
pJQ200SK	Suicide vector for gene replacement, GenR	(32)
pSIJ8	Temperature sensitive plasmid for lambda Red recombinase genes and flippase recombinase expression, AmpR	(33)
pKD4	Template DNA for *kanR* cassette, KmR	(29)
Primers		
pJQ200SK_F	5′-tatggatggtggtgagccagatccttggcgtaatcatggtcatagctgtt	This study
pJQ200SK_R	5′-aatatcgctatgacgcggacggctaagcgcgcaattaaccctcactaaag	This study
srnA_upstream_F	5′-ctttagtgagggttaattgcgcgcttagccgtccgcgtcatagcgatatt	This study
srnA_upstream_R	5′-cgtgcaatccatcttgttcaatcatgactcaccccatcattttaattgac	This study
KanR_F	5′-gtcaattaaaatgatggggtgagtcatgattgaacaagatggattgcacg	This study
KanR_R	5′-atactgggttgtcatcggttactcctcagaagaactcgtcaagaaggcga	This study
srnA_downstream_F	5′-tcgccttcttgacgagttcttctgaggagtaaccgatgacaacccagtat	This study
srnA_downstream_R	5′-aacagctatgaccatgattacgccaaggatctggctcaccaccatccata	This study
srnA_TC_forward	5′-ccccAAGCTTtacgctgaacatcgtacccaacg	This study
srnA_TC_reverse	5′-ccccGAATTCcaggtctttgtaatccttgca	This study
ynfEF_KO_forward	5′-ttcaatatataaacttttatataacgataaagaacagggagtgagttatggtgtaggctggagctgcttc	This study
ynfEF_KO_reverse	5′-atccatattgtgtggtcatgggctactccttaaaccttttcgatctggaccatatgaatatcctccttag	This study

Late exponential cultures in LB broth grown in sealed serum bottles under oxygen-depleted conditions were used as inoculum in selenate reduction assays. For plate assays, a 1 µL aliquot was transferred to LB agar amended with 10 mM sodium selenate. Colonies that tested positive for Se(VI) reduction precipitated red elemental selenium [Se(0)] on the agar, while cells that formed white colonies were defective in Se(VI) reduction. For liquid media assays, cells grown in LB broth were centrifuged and washed with a M9 basal medium composed of Na_2_HPO_4_ (0.68 g/L), KH_2_PO_4_ (0.3 g/L), NaCl (0.05 g/L), NH_4_Cl (0.1 g/L), and MgSO_4_ (0.024 g/L). The cells were then resuspended in the M9 basal medium amended with glucose (4.0 g/L) and Na_2_SeO_4_ (0.25 g/L). Cultures were incubated in sealed serum bottles and sampled at periodic intervals. The aliquots were filtered (0.2 µm) and analyzed for dissolved selenate using an ion chromatography system (Thermo Scientific Dionex Aquion) equipped with an IonPac AS9-HC analytical column (250 mm × 4 mm). The eluent used for the anion ion chromatography analysis was a 9 mM sodium carbonate solution.

### Nucleic acid extraction, sequencing, and annotation

Genomic DNA extraction was performed with Qiagen DNeasy ultraClean Microbial extraction kit. Plasmid extraction was performed using the NEB Monarch Plasmid Miniprep Kit. Nucleotide sequencing was performed by Seqcenter (Pittsburgh, PA, USA) and Plasmidsaurus (Louisville, KY, USA). Quality control checks on raw sequence data were performed using fastQC, and the reads were assembled with SPAdes (34, 35). The assembled contigs were analyzed by using RAST (36). Database searches were conducted with identified ORFs by using the BLAST algorithm (www.ncbi.nlm.nih.gov/BLAST). Subsequent filtering of the RAST contigs through the NCBI submission portal yielded a final set of 142 contigs. These were further annotated using Prokka (37). Profile HMM Searches were conducted using HMMER3 (38) against the Pfam database (39) to identify proteins with the Pfam domain for molybdopterin dinucleotide binding (PF01568). Signal peptide prediction was performed using the SignalP 6.0 web server (40).

### Mutagenesis of the *srnA* gene in *E. cloacae*

Gene knockout mutant strains were constructed using the *sacB*-based vector pJQ200SK (32). Four sets of primer pairs were designed: (i) srnA_upstream_F and srnA_upstream_R, (ii) srnA_downstream_F and srnA_downstream_R, (iii) KanR_F and KanR_R, and (iv) pJQ200SK_F and pJQ200SK_R. The PCR primers were used to amplify approximately 1,000-bp upstream and downstream sequences of the *srnA g*ene and to incorporate a *kanR* cassette between the flanking sequences. The pJQ200SK plasmid was linearized, and the PCR products were joined together using Gibson assembly. The construct was then mated into *E. cloacae* SLD1a-1 using *E. coli* S17-1 as the donor strain. Homologous recombination resulted in the integration of the *kanR* cassette into the *srnA* ORF. Transconjugants were incubated anaerobically overnight in LB containing the appropriate antibiotics and subsequently transferred to anoxic LB broth containing 5% sucrose and kanamycin. Mutants that had undergone a second recombination event were plated on LB agar containing 10 mM sodium selenate. Colonies that appeared white on the agar plate had lost the ability to reduce Se(VI) to red Se(0) and were selected for genome sequencing to verify that the selenate reductase gene *srnA* was replaced by the *kanR* cassette.

### Deletion of the *ynfEF* genes in *E. coli*

The wild-type *E. coli* strain reduced Se(VI), and in order to conduct the heterologous expression experiment, the native selenate reductase genes in *E. coli* were deleted. Deletion of the *ynfEF* selenate reductase gene pair in *E. coli* BW25113 was performed using lambda Red recombineering with the pSIJ8 plasmid (29, 33). First, PCR primers were designed to amplify the FRT-flanked *kanR* gene in pKD4 (Table 1). The forward and reverse primers contained oligonucleotides with 50-bp homology to the *ynfE* start codon and *ynfF* stop codon, respectively. The PCR product was then transformed into *E. coli* BW25113 containing the pSIJ8 plasmid. *E. coli* cells washed with calcium chloride were subjected to heat shock for the transformation. After heat shock, cells were added to 1 mL LB and incubated for 2 h at 37°C, allowed to recover overnight at room temperature, and plated onto LB agar to select for KmR transformants. Once mutant strains were obtained, the *kanR* cassette was removed using L-rhamnose induction at 30°C. Deletion of *ynfEF* was verified by colony PCR using Q5 2 × Master Mix (New England Biolabs) according to the manufacturer’s instructions. The pSIJ8 plasmid was cured from the cells by growing cultures at 37°C.

### Complementation experiments with psrnA

The *srnA* gene was cloned for complementation of *E. cloacae* and *E. coli* mutants that had lost the ability to reduce selenate. The primer set srnA_TC_forward containing a HindIII site and srnA_TC_reverse containing an EcoRI site was used to amplify and clone the *srnA* gene in the *E. cloacae* wild-type strain (Table 1). The PCR product and the plasmid pRK415 were digested using the restriction enzymes EcoRI and HindIII and then ligated together, resulting in the plasmid construct psrnA. The plasmid was mobilized into the *E. cloacae* Δ*srnA* mutant by conjugation using *E. coli* S17-1 as the donor strain. The plasmid was also transferred into the *E. coli* Δ*ynfEF* mutant by transformation using the heat-shock method. The *E. cloacae* and *E. coli* transconjugants were selected using the appropriate antibiotics and then screened for Se(VI) reduction activity on selenate-containing LB agar.

### Protein structure modeling

The selenate reductase protein structure model was generated using Boltz-1 (41) and AlphaFold3 (42). The amino acid sequences of the three protein subunits—SrnA (molybdenum-containing catalytic subunit), SrnB (iron-sulfur cluster-binding subunit), and SrnC (membrane anchor)—were supplied in FASTA format from RAST annotations. Iron-sulfur clusters were specified using the Chemical Component Dictionary code “SF4” (43), and the bis(molybdopterin guanine dinucleotide)molybdenum(IV) cofactor was included using its SMILES string (44). To help identify the location and structural features of the active site in SrnA and to explore plausible binding modes of the selenate substrate within this site, the selenate anion was included in the input using CCD code “SE4.” The Boltz-1 model was used to investigate how the three subunits assembled into a functional complex, to localize each cofactor within its respective subunit, and to examine the surface architecture of the catalytic site in SrnA. In the AlphaFold3 model, diffusion sampling was performed using fixed random seeds representing a limited sampling regime rather than exhaustive conformational exploration. Among the protein structures produced, the top-ranked prediction based on the default AlphaFold3 ranking metric was selected for further analysis. Molecular graphics and analyses were performed in UCSF ChimeraX (45). The MatchMaker tool was used to align the AlphaFold3 and Boltz-1 models and compute the root-mean-square deviation (RMSD) between matching atoms. The resulting RMSD values were low across all subunits.

### Phylogenetic analysis

We constructed a multiple sequence alignment of SrnA, SerA, and 21 other related proteins (Table S2) using MUSCLE on Seaview (46). Maximum likelihood phylogeny trees were inferred from the alignment using PhyML (47) with the Le and Gascuel amino acid replacement matrix (48).

## Data Availability

The nucleotide sequence of the *srnABCD* operon was deposited in GenBank under accession number PX138932. The genome sequence is available in NCBI under accession PRJNA1345729. The raw sequencing data are available in the NCBI Sequence Read Archive (SRA) under accession SRX30857568.

## SUPPLEMENTAL MATERIAL

Supplemental material aem.01796-25-s0001.pdf Tables S1 and S2; Fig. S1 and S2.

ASM does not own the copyrights to Supplemental Material that may be linked to, or accessed through, an article. The authors have granted ASM a non-exclusive, world-wide license to publish the Supplemental Material files. Please contact the corresponding author directly for reuse.
