# Supplementary material for: Genetic identification of the selenate reductase in Enterobacter cloacae SLD1a-1
Source: Appl Environ Microbiol. 2025 Nov 26;91(12):e01796-25. doi: 10.1128/aem.01796-25 (PMC12724226; doi:10.1128/aem.01796-25)
Supplement: Supplemental material — Tables S1 and S2; Fig. S1 and S2. [file aem.01796-25-s0001.pdf]

Table S1. Genome properties of *E. cloacae* SLD1a-1

**Attribute**	**Value**
Genome size (bp)	4,874,235
DNA coding (bp)	4,344,897
DNA G + C (bp)	2,675,719
DNA scaffolds	142
Total genes	4,601
Protein coding genes	4,518
RNA genes	83
Genes with function prediction	3,360
Genes assigned to COGs	3,922
Genes with Pfam domains	4,199
Genes with molybdopterin binding Pfam domain	13
Genes with signal peptides	571
Genes with TAT signal peptides	19
Genes with transmembrane helices	1,435
Number of plasmids	0

Table S2. Representative proteins related to SrnA and SerA used in the multiple sequence alignment and construction of the phylogenetic tree

Protein	Organism	Genbank ID	Uniprot ID
SerA	*Thauera selenatis*	CAB53372.1	Q9S1H0
ClrA	*Ideonella dechloratans*	CAD97447.1	P60068
DdhA	*Rhodovulum sulfidophilum*	AAN46632.1	Q8GPG4
EbdA	*Azoarcus sp. EB1*	AAK76387.1	Q93PD2
NarG	*Pseudomonas fluorescens*	AAG34373.1	Q9F0X7
NarG	*Bacillus subtilis (strain 168)*	CAB15756.2	P42175
NarG	*Escherichia coli (strain K12)*	CAA34303.1	P09152
NarG	*Mycobacterium tuberculosis*	CCP43917.1	P9WJQ3
BisC	*Escherichia coli*	AAC76575.3	P20099
BisC	*Cereibacter sphaeroides (Rhodobacter)*	AAA74739.1	P54934
DorA	*Rhodobacter capsulatus*	AAD13674.1	Q52675
DorA	*Cereibacter sphaeroides (Rhodobacter)*	AAB94874.1	O30744
TorA	*Escherichia coli*	AAC74082.1	P33225
TorA	*Shewanella massilia*	CAA06851.1	O87948
TorA	*Shewanella putrefaciens*	CAA06794.1	O86914
DmsA	*Escherichia coli*	AAC73980.2	P18775
DmsA	*Haemophilus influenzae*	AAC22706.1	P45004
YnfE	*Citrobacter freundii*	AFI57880.1	I1W1S3
YnfE	*Escherichia coli*	AAC74659.1	P77374
YnfF	*Escherichia coli*	AAC74660.4	P77783
SrnA	*Enterobacter cloacae*	XZP31502.1	N/A
YnfE	*Salmonella enterica*	ADX17198.1	E8XI79
YnfF	*Salmonella enterica*	ADX17197.1	E8XI78

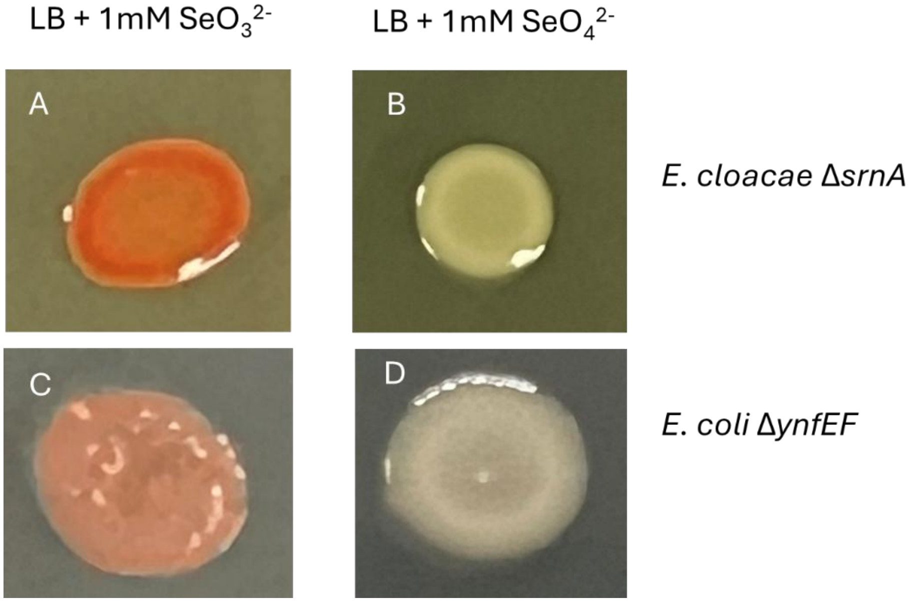

Figure S1. Selenite (SeO_3^{2-}) and selenate (SeO_4^{2-}) reduction by the *E. cloacae* Δ*srnA* and *E. coli* Δ*ynfEF* mutant strains. A) Red elemental selenium was formed when the Δ*srnA* mutant strain was grown on LB containing 1 mM selenite due to the reduction of Se(IV) to Se(0). Mutation of *srnA* did not affect Se(IV) reduction activity; B) No Se(0) was formed when the Δ*srnA* mutant strain was grown on LB containing 1 mM selenate. Mutation of *srnA* abolished selenate reduction activity and the mutant strain was unable to convert Se(VI) to Se(IV). C) The *E. coli* Δ*ynfEF* mutant strain formed pink colonies on selenite-containing LB agar due to the precipitation of elemental selenium; D) The *E. coli* Δ*ynfEF* mutant strain formed white colonies on selenate-containing LB agar and was unable to reduce Se(VI).

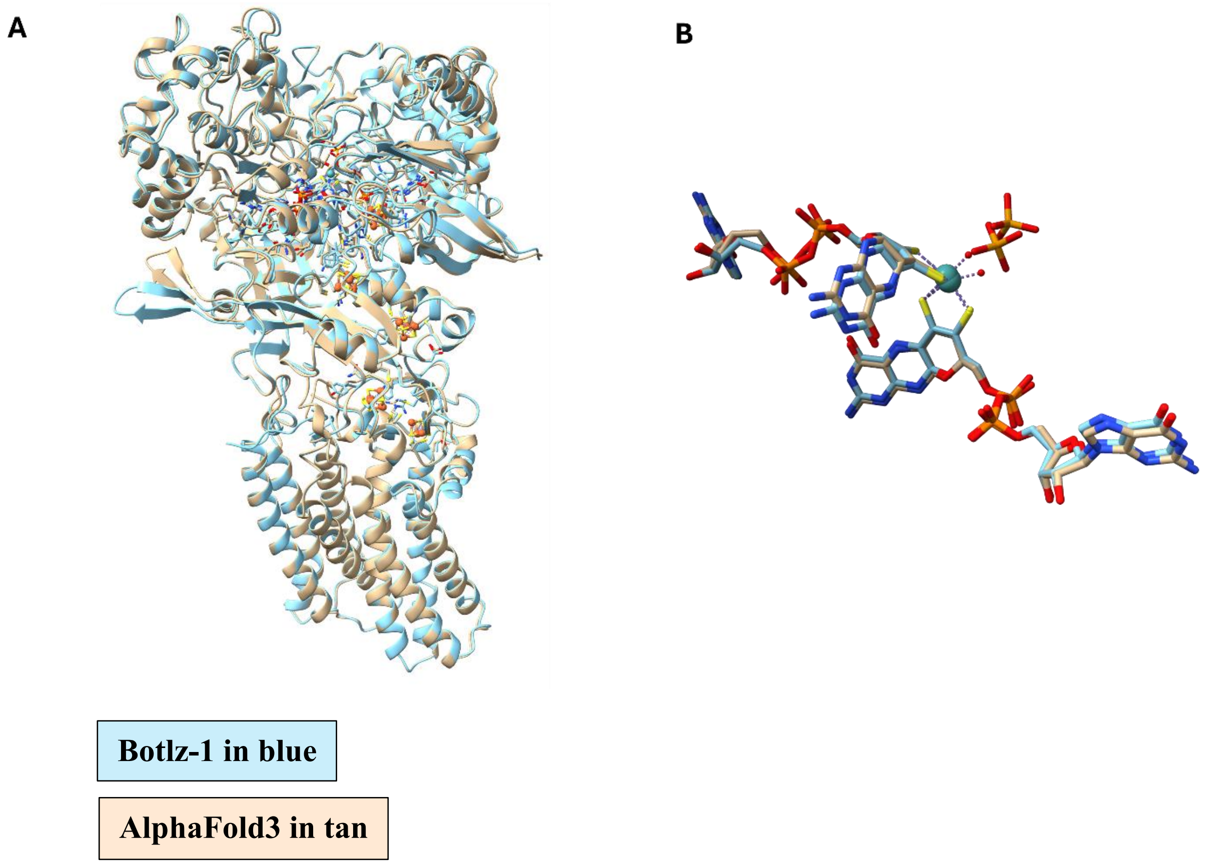

Figure S2. A comparison between the Boltz-1 and AlphaFold3 structures. A) Overlay of the two protein structures, and B) Superposition of the molybdopterin cofactor. The models converge in atomic detail on the same protein domain arrangement and cofactor configurations. The root-mean-square deviation between matching atoms were low across all subunits.
